# A Google Trends Approach to Identify Distinct Diurnal and Day-of-Week Web-Based Search Patterns Related to Conjunctivitis and Other Common Eye Conditions: Infodemiology Study

**DOI:** 10.2196/27310

**Published:** 2022-07-05

**Authors:** Michael S Deiner, Gurbani Kaur, Stephen D McLeod, Julie M Schallhorn, James Chodosh, Daniel H Hwang, Thomas M Lietman, Travis C Porco

**Affiliations:** 1 Francis I Proctor Foundation University of California San Francisco San Francisco, CA United States; 2 Department of Ophthalmology University of California San Francisco San Francisco, CA United States; 3 School of Medicine University of California San Francisco San Francisco, CA United States; 4 Department of Ophthalmology Harvard Medical School Boston, MA United States; 5 Massachusetts Eye and Ear Harvard Medical School Boston, MA United States; 6 Stanford University San Mateo, CA United States; 7 The Nueva School San Mateo, CA United States; 8 Department of Epidemiology and Biostatistics University of California San Francisco San Francisco, CA United States; 9 Global Health Sciences University of California San Francisco San Francisco, CA United States

**Keywords:** diurnal eye conditions, hebdomadal, online search, web-based search, eye conditions, infodemiology, dry eye, conjunctivitis, pink eye, information seeking, vision

## Abstract

**Background:**

Studies suggest diurnal patterns of occurrence of some eye conditions. Leveraging new information sources such as web-based search data to learn more about such patterns could improve the understanding of patients’ eye-related conditions and well-being, better inform timing of clinical and remote eye care, and improve precision when targeting web-based public health campaigns toward underserved populations.

**Objective:**

To investigate our hypothesis that the public is likely to consistently search about different ophthalmologic conditions at different hours of the day or days of week, we conducted an observational study using search data for terms related to ophthalmologic conditions such as conjunctivitis. We assessed whether search volumes reflected diurnal or day-of-week patterns and if those patterns were distinct from each other.

**Methods:**

We designed a study to analyze and compare hourly search data for eye-related and control search terms, using time series regression models with trend and periodicity terms to remove outliers and then estimate diurnal effects. We planned a Google Trends setting, extracting data from 10 US states for the entire year of 2018. The exposure was internet search, and the participants were populations who searched through Google’s search engine using our chosen study terms. Our main outcome measures included cyclical hourly and day-of-week web-based search patterns. For statistical analyses, we considered *P*<.001 to be statistically significant.

**Results:**

Distinct diurnal (*P*<.001 for all search terms) and day-of-week search patterns for eye-related terms were observed but with differing peak time periods and cyclic strengths. Some diurnal patterns represented those reported from prior clinical studies. Of the eye-related terms, “pink eye” showed the largest diurnal amplitude-to-mean ratios. Stronger signal was restricted to and peaked in mornings, and amplitude was higher on weekdays. By contrast, “dry eyes” had a higher amplitude diurnal pattern on weekends, with stronger signal occurring over a broader evening-to-morning period and peaking in early morning.

**Conclusions:**

The frequency of web-based searches for various eye conditions can show cyclic patterns according to time of the day or week. Further studies to understand the reasons for these variations may help supplement the current clinical understanding of ophthalmologic symptom presentation and improve the timeliness of patient messaging and care interventions.

## Introduction

Infodemiology is a relatively young discipline within health informatics studying the science of distribution and determinants of information within an electronic medium, specifically the internet or in a population, with the aim of informing public health and policy [1-3]. Applications of this form of health informatics have included predicting coronavirus outbreaks based upon queries of web-based search engines, syndromic surveillance by analysis of status updates or tweets on Twitter, tracking the disparities in access to health care information, and mining search engine data to cluster query click data to estimate prevalence of certain conditions that patients seek to address themselves outside of clinical settings or hours or to study prevalence of factors in typically unobserved locations [2,4-6]. One key advantage proffered by these approaches to public health analytics compared to collating and probing large data sets is the ability to conduct real time predictive analysis of health-related behaviors [2,7,8]. For example, one study found that the number of clicks on a keyword-triggered link in Google demonstrated a strong correlation with the following week of influenza cases during the 2004-2005 Canadian influenza season [9]. Similarly, another study found that social media–based surveillance for foodborne diseases were 66% as effective, rapid, and cheaper than standard database surveillance systems [10].

Google Trends has become a popular tool for infodemiologic studies in predicting disease occurrence and outbreaks, so much so that standardized approaches seeking to strengthen validity of such analyses have been proposed, and commonly used data access tools have been developed [11,12]. However, limitations when using Google Trends must also be considered. For example, for COVID-19, media coverage can affect web searches [13,14], and search volume values can vary depending on the date of data collection [15,16]. Applications in this field are vast (eg, use of Google Trends for public health planning regarding marginalized populations or birth control, to name a few [17,18]) and can adapt rapidly to current events [3]. Recent Google Trends studies have explored, for example, the potential impact of the COVID-19 pandemic on mental health behavior and child mistreatment [19-22] on ocular and other communicable and noncommunicable disease [23,24] and on treatment and misinformation related to COVID-19 itself [25-27].

Cyclic patterns of Google Trends search interest as related to human health, often seasonal but also to a letter extent diurnal, are an area of extensive research. Clinical study has identified cyclic occurrence of health conditions in humans, including diurnal eye-related conditions, and the results may facilitate chronopreventive and chronotherapeutic care [28-35]. Web-based search behavior regarding nonocular disease symptoms has been shown to reflect seasonal and diurnal clinical cyclicity as well as aspects of disease not typically observed in clinics at all (for example, coronary heart disease and depression) [36,37]. Web-based search or social media data also can reflect seasonal or emerging clinical eye disease patterns and conjunctivitis epidemics on relatively long timescales, including the impact of other factors such as the COVID-19 pandemic [8,23,38-43]. This suggested that, as with other health conditions [36,37], there is the potential to add to our knowledge about diurnal and day of week aspects of eye disease outside of the days and times that patients are typically seen in clinics, using web-based hourly search data. Herein, we tested the hypothesis that the public is likely to search about different aspects of eye health at different (but predictable) hours of the day or days of week. Specifically, we conducted an observational study investigating if US hourly web-based search data for terms related to conjunctivitis or other common eye conditions and treatments could demonstrate diurnal or day-of-week cyclic patterns and if those patterns were distinct from each other. For example, daily occurrence peaks may occur at different times, or the difference between the peak and the trough may differ.

## Methods

### Google Search Data

We queried Google Trends for conjunctivitis terms and other common eye conditions and treatments for comparison. Search terms included “conjunctivitis,” “blurry eyes,” “cataracts,” “pink eye,” “dry eyes,” “watery eyes,” “glaucoma,” “contact lenses,” “visine,” and “lasik.” A positive control term that would likely exhibit hourly and day-of-week variation (“drunk”) was included. Data were obtained using a Python (Python Software Foundation) script we developed to apply using *Pytrends* (a commonly used application programming interface to access Google Trends data) to obtain Historical Hourly Interest data, using the pytrends.get_historical_interest application programming interface [4,6,12]. Each term and state combination were queried individually. Each request retrieved 1 week of hourly data. The results were combined for analysis. Using this method, no categories were specified in the query, quotes were not used, and terms were queried individually as terms and not as *topics*. Relative search volume (RSV) of hourly search frequency data for these terms for the year 2018 (the most recent complete year of data available at the time of our query) from the 10 most populous US states (CA, FL, GA, IL, MI, NC, NY, OH, PA, and TX) were downloaded. Data were queried and downloaded twice for each state-term pair to account for random sampling during the week of August 26, 2019 [36,37]. Universal Coordinated Times were adjusted to the predominant time zone for each state (only FL, MI, and TX include multiple time zones). The resulting time series represented hourly RSV for a given location, time period, and term. Data for all states were then combined for analysis.

### Diurnal and Day of Week Analysis and Comparison of Cyclic Strength and Peak Times

Using the hourly RSV for each search term as an outcome variable, we conducted Serfling regression adjusting for trend, as follows [44-46]. We adjusted for overall trend using third-order orthogonal polynomials in the number of days since January 1, 2018. Diurnal effects were modeled by terms of the form sin *nωt* and cos *nωt*, where *ω=2π/24, t* is the time measured on a 24-hour clock, and *n*=1,…,4. We estimated separate diurnal effects for weekend days and for weekdays.

Because an outlier occurring at a single time could produce biased estimates of diurnal coefficients, we include additional terms to control for potential outliers (nuisance terms). We potentially include a large number of such terms of varying lengths, avoiding nonidentifiability by use of cross-validated LASSO (least absolute shrinkage and selection operator) to select only a small number of such terms [47]. This provides a simple regression-based filter for removing apparent epidemics and other irregular outliers. Specifically, outliers and localized (nonperiodic) departures were modeled by terms that take the value 1 on given intervals and are 0 otherwise. Specifically, we chose terms of the form 1*x*∈*[km,k(m+1)-1)*, where *m*=0,1,…, *x* is the number of hours elapsed since midnight, January 1, 2018, and *k* takes values 8, 16, 32, 64, and 128, as well as 168 (the latter corresponding to the number of hours in a week). We also chose other intervals in a sensitivity analysis, finding that the choice of these regressors had little effect on the results; specifically, we chose the set k=7, 14, 28, 56, 112, and 168 hours, as well as the set 9, 18, 36, 72, 144, and 168 hours. Other choices for filtering outliers could have been chosen instead of this regression procedure.

For statistical analyses, following model selection for these nuisance terms, ordinary least squares estimation was used to estimate the trend, outlier, and trigonometric coefficients. From the trigonometric coefficients and intercept, we estimated the circular median occurrence time and the amplitude-to-mean ratio (in a similar fashion as our previous analyses and using the R [R Foundation for Statistical Computing] package “circular”) [39-41]. Because diurnal and day of week occurrence data are angular data, we used the circular median time to summarize the central tendency; the circular median reflects the peak occurrence (when the data are approximately unimodal). The amplitude-to-mean ratio measures the cyclicity, with values near zero indicating small cyclic variability. Standard errors and *P* values were determined using time series bootstrap, with a fixed width of 20 hours [48-50]. For diurnal cyclic patterns, *P* values less than .001 were considered significant.

**Figure 1:**
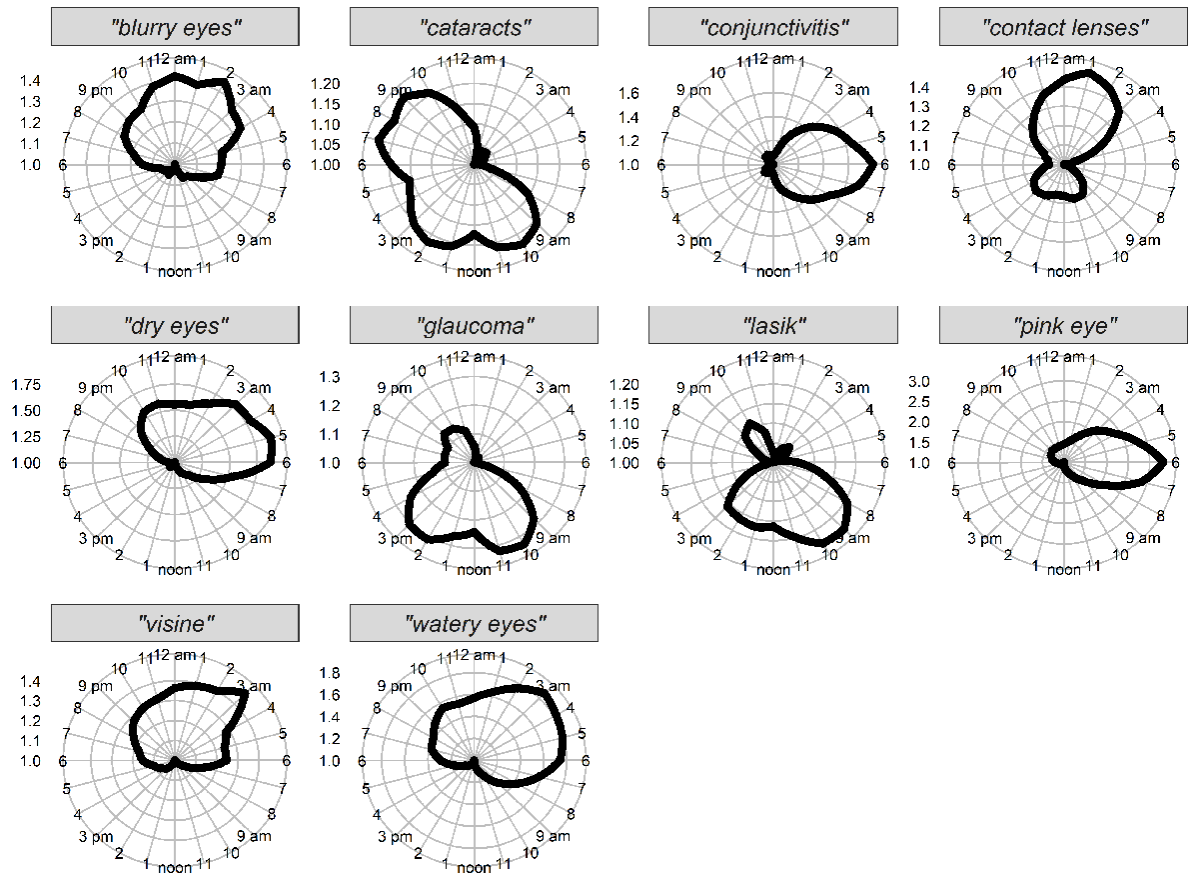
Average hourly cyclic pattern from 2018 for 10 US states combined.

**Figure 2:**
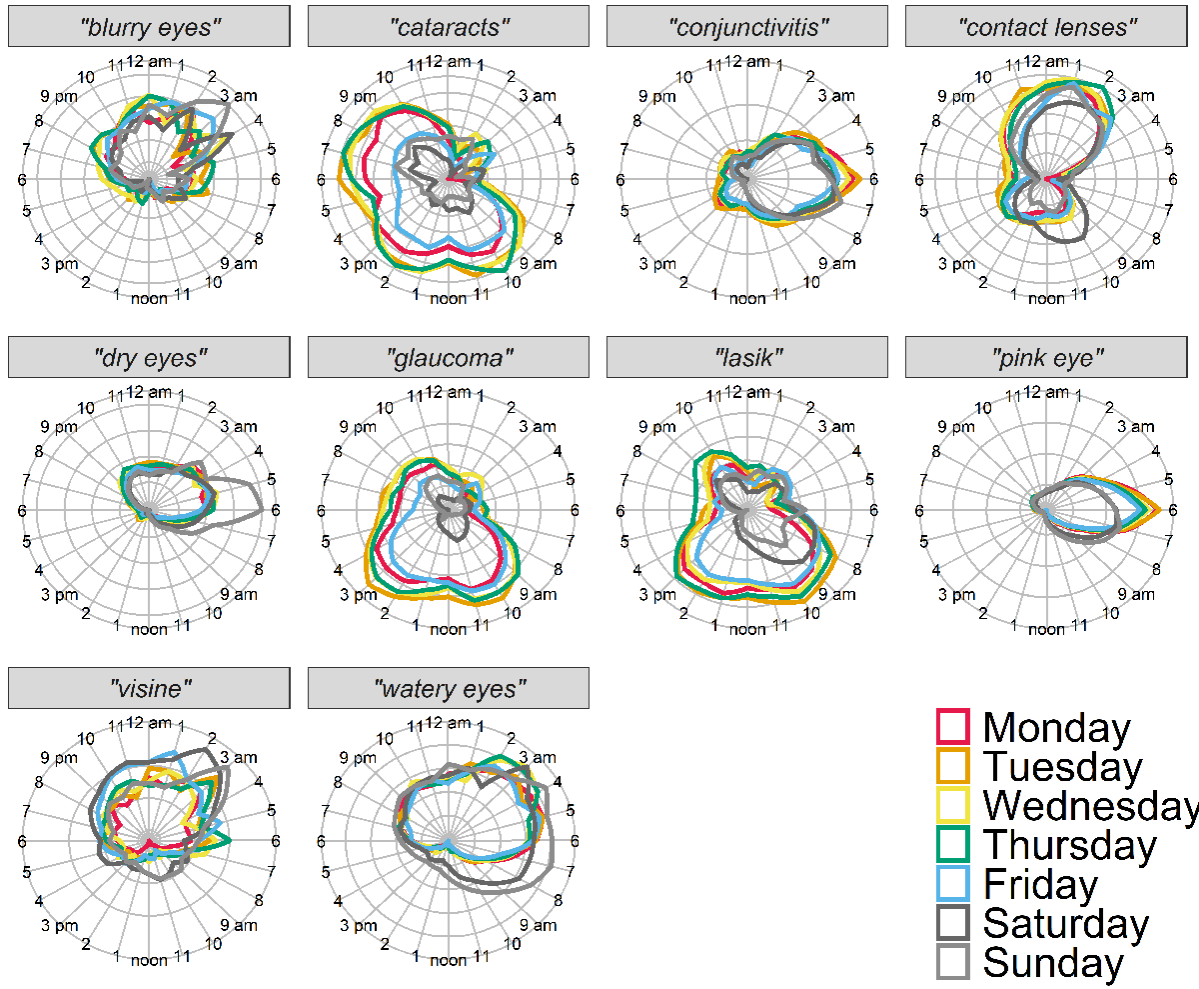
Average hourly cyclic pattern per weekday from 2018 for 10 US states combined.

**Figure 3:**
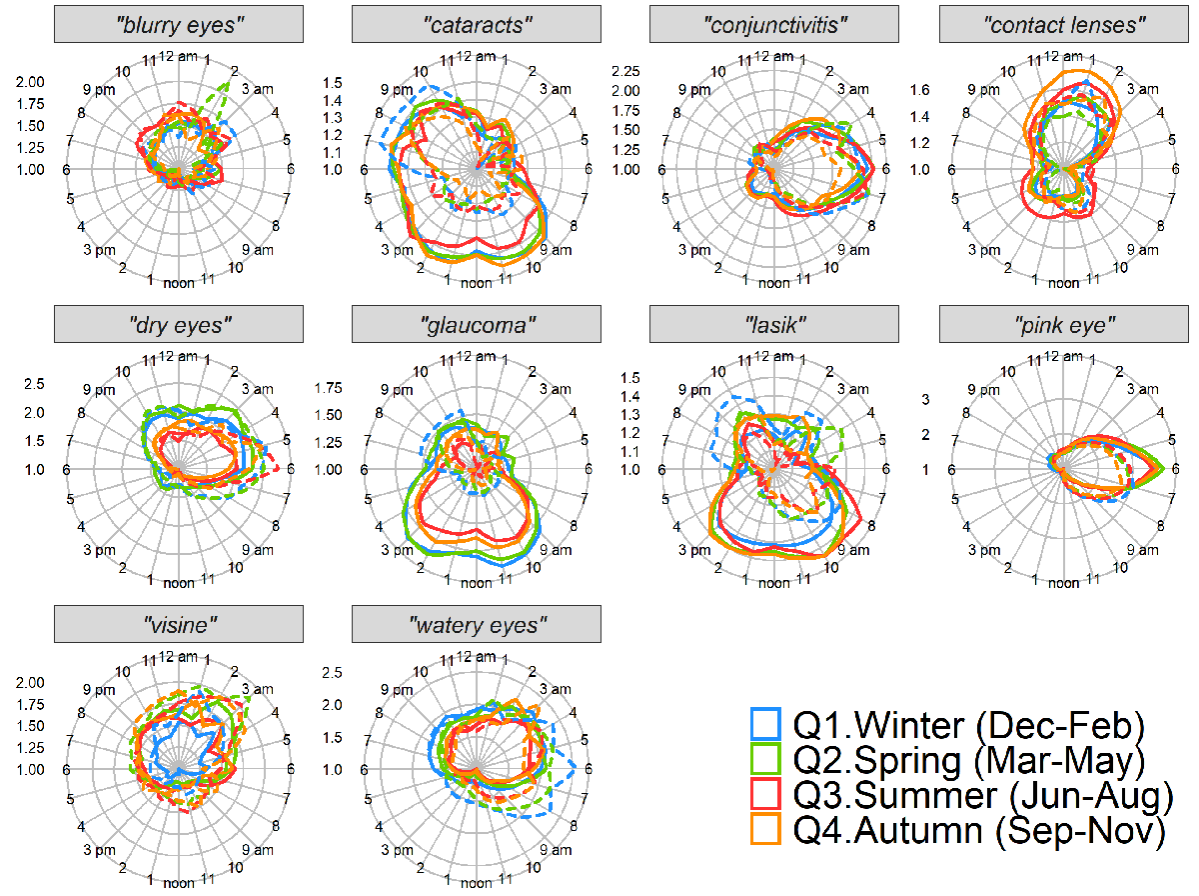
Average hourly cyclic pattern per season for weekdays (solid) and weekend days (dashed) from 2018 for 10 US states combined.

For data visualizations, mean hourly results of the filtered time series data for each term were normalized for visual comparison in polar plots (R package “ggplot” [51]). In order to optimally demonstrate cyclic patterns per terms in the plots, hourly RSVs for each term were normalized by dividing the mean per each hour per term by the value of the hour having the smallest mean value such that the hours with the least RSV are plotted closest to the center with a value of 1.0, while hours of higher relative search interest were plotted further from the center. Since values have been normalized, plots do not represent total search interest for one term vs another—but instead represent the relative amount of search interest between terms (Figure 1), between days for an individual term (Figure 2), or between seasons and weekday vs weekend day for an individual term (Figure 3).

### Ethics Approval

University of California San Francisco Institutional Review Board approval (14-14743) was obtained for this study.

## Results

Overall, we found that each search term exhibited cyclic diurnal patterns of search interest (*P*<.001 for all terms). However, cyclic strength and central tendency differed between search terms, as described below.

### Hourly, Weekly, and Seasonal Patterns

To visualize cyclic diurnal patterns for each term, mean RSV at each time of day is represented in normalized polar 24-hour plots (see Methods) in Figure 1. Note that despite most terms exhibiting diurnal patterns, scale bars in Figure 1 indicate that not all terms exhibited similar diurnal strength. In Figure 2, cyclic diurnal patterns for each term on each day of the week are presented as normalized polar 24-hour plots. These plots suggested some terms had diurnal cyclic features with patterns that varied between weekdays and weekends. Terms shown to have statistically significant day-of-week patterns, mean peak day values, and other day-of-week characteristics for all terms are shown in Table 1. In Figure 3, normalized polar 24-hour plots indicate cyclic diurnal patterns for each term for each season for weekday and weekends. Weekday group results are shown as solid lines, and weekend day group results as dashed lines. Seasons are indicated by color. These plots suggested that although most terms had similar diurnal and weekday search patterns per season, in some cases, features varied by season. For example, “dry eyes” tended to have more RSV overall throughout the hours of winter and spring, but also exhibited a strong morning peak seen in summer weekends, as did “watery eyes” in winter and spring weekends.”

**Table 1:** Cyclical diurnal or day-of-week characteristics of relative search values.

Search terms	Circular median times^a^				Amplitude-to-mean ratio		
	Weekday	Weekend	Difference, hrs	Weekday^b^		Weekend	Difference^c^
“blurry eyes”	00:39 (00:18, 00:59)	01:09 (00:40, 01:37)	0-2	0.37 (0.33, 0.47)		0.42 (0.39, 0.59)	0.02 (–0.04 to 0.08)
“cataracts”	15:28 (15:11, 15:45)	18:35 (17:12, 20:15)	2-4	0.26 (0.24, 0.3)		0.18 (0.15, 0.25)	–0.05 (–0.08 to –0.02)
“conjunctivitis”	06:11 (06:02, 06:21)	05:54 (05:46, 06:03)	0-2	0.62 (0.59, 0.66)		0.77 (0.72, 0.84)	0.04 (0 to 0.07)
“contact lenses”	23:16 (22:59, 23:32)	02:29 (01:57, 03:02)	2-4	0.43 (0.41, 0.47)		0.38 (0.35, 0.43)	–0.03 (–0.06 to –0.01)
“dry eyes”	02:13 (02:07, 02:20)	03:38 (03:28, 03:48)	0-2	0.61 (0.58, 0.66)		0.85 (0.79, 0.95)	0.12 (0.08 to 0.17)
“glaucoma”	13:24 (13:14, 13:34)	00:13 (21:41, 02:52)	4-6	0.38 (0.36, 0.41)		0.15 (0.12, 0.19)	–0.15 (–0.17 to –0.13)
“lasik”	11:51 (11:33, 12:09)	06:00 (05:10, 06:51)	4-6	0.28 (0.25, 0.31)		0.24 (0.2, 0.28)	–0.03 (–0.06 to –0.01)
“pink eye”	04:35 (04:33, 04:38)	05:38 (05:33, 05:43)	0-2	1.6 (1.56, 1.64)		1.08 (1.04, 1.11)	–0.26 (–0.29 to –0.24)
“visine”	00:37 (00:17, 00:56)	00:37 (00:08, 01:04)	0-2	0.41 (0.35, 0.5)		0.43 (0.38, 0.55)	0.03 (–0.03 to 0.09)
“watery eyes”	01:49 (01:41, 01:56)	03:50 (03:35, 04:07)	2-4	0.67 (0.63, 0.74)		0.48 (0.45, 0.58)	–0.07 (–0.12 to –0.03)

^a^The average filtered and detrended circular median time (and 95% CI) for each term for weekdays and weekend days. We found evidence that the CIs of the coefficients measuring diurnality excluded zero, indicating statistically significant diurnal variation (*P*<.001 for all values).

^b^Peak-to-trough divided by mean value to normalize the scalar difference. A larger average daily amplitude-to-mean ratios value indicates a more pronounced diurnal pattern.

^c^Allows a comparison of weekday to weekend diurnal cycle amplitude-to-mean ratios (ie, a comparison of cyclic strengths), providing the average difference (and 95% CI) between weekday vs weekend amplitude-to-mean ratios. Negative values indicate stronger weekday cyclic strength, and positive values indicate stronger weekend cyclic strength. Values further from 0 indicate a larger difference between weekdays and weekend days. This column is a difference in amplitudes divided by the average of the weekend and weekday means, not a difference between the previous two columns.

### Statistical Analysis of Cyclic Patterns

Following smoothing and detrending, the resulting data set was used for all subsequent statistical analyses and data visualizations. The results for all terms are presented in Table 1. Columns 2-3 provide weekday and weekend circular median times, and column 4 provides differences between weekday and weekend for the peak times. Columns 5-6 provide amplitude-to-mean ratios. Column 7 provides a comparison of weekend to weekday cyclic ratios (values further from zero indicate larger differences, negative values indicate stronger weekday cyclic strength, and confidence intervals crossing zero indicate no significant difference).

For all terms, diurnal cyclic patterns were significant on weekdays and on weekends (*P*<.001 for all terms). Characteristics differed by search terms. Of the eye-related terms, “pink eye” had the strongest diurnal cyclic patterns based on amplitude-to-mean ratios, with stronger signal restricted to a narrow time window and peaking all mornings within the same 1-hour period. This showed a higher amplitude on weekdays (Table 1, columns 5-7; Figures 2 and 3). “Conjunctivitis” also had one of the stronger diurnal cyclic patterns, but lower than “pink eye,” with a slightly later morning circular median time and less cyclic strength difference between weekend and weekday. In contrast to “pink eye,” “dry eyes” exhibited a stronger diurnal pattern on weekends, with stronger signal occurring over a broader evening-to-morning time window, peaking in early morning and most significantly on Sunday mornings (Table 1, columns 5-7; Figure 2 and 3). Similar to “pink eye” though, “dry eyes” circular median times were nearby on weekday compared to weekend. By contrast, weekday vs weekend circular median times for “cataracts,” “glaucoma,” and “lasik” were less aligned, and weekday RSV was larger than weekend overall for these terms (Table 1, column 4; Figures 2 and 3). As a positive control, the term “drunk” exhibited a strong amplitude-to-mean ratio that was strongest from late evening through early morning on weekends (data not shown), reflecting late-evening alcohol consumption.

## Discussion

### Principal Findings

Web-based search behavior patterns for terms related to common eye conditions and treatments exhibited significant unique cyclic diurnal variation. This suggests that leveraging infodemiological approaches such as those demonstrated in this study can add information to our understanding of the times of day and night when different ocular conditions may be of the most or least perturbance or concern to patients. This may help augment our traditional understanding of ocular conditions, which has been based predominantly upon assessing patients with ophthalmology conditions during typical clinic hours. We observed that features occurring outside of typical clinic hours can differ between ophthalmologic condition–related search terms. For example, “pink eye” showed larger diurnal amplitude-to-mean ratios over a short daily weekday morning time period, while other terms such as “dry eyes” had a larger amplitude diurnal pattern on weekends, with stronger signal occurring over a broader evening-to-morning period compared to “pink eye.”

For some individual search terms, we also found significant differences in diurnal search patterns on weekdays vs weekends for that term. This suggests infodemiological approaches can provide new understanding of specific days of the week, and hours of those days, on which particular ophthalmologic conditions are most affecting patients. Such approaches can add to the ongoing research studies to understand critical times or days for severity or treatment of symptoms and conditions outside of standard clinic hours for ocular conditions, as has also been studied for other disease [28,31-37,52-57]. We also observed that diurnal search patterns can differ by time of year for some terms, suggesting unique seasonal factors may affect the diurnal cycle of specific ocular conditions and raising the potential value of the approaches such as ours for enhancing the study of seasonal eye disease [29].

### Limitations

Our study has potential for outliers, bias, and confounders. For example, an isolated event, such as a celebrity contracting conjunctivitis, could trigger an unusual search for “pink eye” at the time that the news story was reported. Similarly, an event such as a power outage could trigger temporary changes in search patterns. Furthermore, it is known that media coverage can impact search for COVID-19–related terms [13,14], and daily RSV can vary with data collection date [15,16]. To account for such aberrancies, we used a model with a regression-based filter to remove unusual surges or decreases such as isolated events and other irregular outliers (see Methods) and used averages from repeated queries and from multiple states and days to reduce potential imprecision. The results appeared stable in sensitivity analyses of the model. Although not incorrect, our approach used search keyword terms and did not allow query of search topics or health category; thus, the study of our health topics may not be fully complete. Future approaches comparing results from multiple years, and optimally using search topics and health categories for refinement in preliminary and final analyses, could provide additional model validation. Future applications of machine learning also has potential to improve the sensitivity and specificity of our model [11,58].

Despite these limitations, we found evidence in support of our model. For example, the results identified in our analyses often reflected components of known clinical understanding. The observed increase in hourly RSV from late night to early morning for “dry eyes” and “blurry eyes” is consistent with clinical reports of the symptoms [31,34]. Stronger amplitude for “dry eyes” observed on weekend mornings might represent elevated prior evening exposure to irritants such as smoke or alcohol, which have been reported to increase these symptoms [32,33]. The observed increase in hourly RSV from evenings to early mornings for “blurry eye,” “contact lenses,” “visine,” and “watery eye” may reflect increased evening and nighttime symptoms of contact lens wearers [35]. Similarly, observed increases in hourly RSV in mornings for “conjunctivitis” and “pink eye” may reflect clinical findings as well [28]. In comparison, diurnal search for “cataracts,” “glaucoma,” and “lasik” occurred more during weekdays at daytimes. This suggests information-seeking behavior related to ocular procedures or chronic conditions not associated with acute symptoms may be more likely to occur during the regular workday.

### Conclusions

In this study, we establish evidence from web-based hourly search patterns that suggest there are distinct diurnal and weekly patterns undergirding web-based information-seeking behavior related to a variety of ophthalmologic symptoms and conditions. More precise temporal understanding of clinical eye disease presentation, hygiene, and health maintenance behaviors among patients outside of the clinic may be ascertained in the future through analysis of complementary data sources such as using web-based search data. This in turn could lead to improved approaches for diurnal eye disease monitoring and timing of resource allotment for ocular telemedicine, timely health care messaging, and clinical interventions.

## Abbreviations

LASSO: least absolute shrinkage and selection operator
RSV: relative search volume
